# Consultation Management during the COVID-19 Pandemic: The Experience of Lithuanian Physicians

**DOI:** 10.3390/healthcare10122472

**Published:** 2022-12-07

**Authors:** Aida Budrevičiūtė, Gediminas Raila, Renata Paukštaitienė, Leonas Valius, Marios Argyrides

**Affiliations:** 1 Independent Scientist, Chief Researcher of the Biomedical Study “Challenges of COVID-19 Pandemic in Family Medicine”, LT-06256 Vilnius, Lithuania; 2 Department of Family Medicine, Kaunas, Lithuanian University of Health Sciences, LT-44307 Kaunas, Lithuania; 3 Department of Physics, Mathematics, and Biophysics, Medical Academy, Lithuanian University of Health Sciences, LT-44307 Kaunas, Lithuania; 4 School of Health Sciences, Neapolis University Pafos, Paphos 8042, Cyprus

**Keywords:** primary health care, family medicine, COVID-19 pandemic, consultation management, Lithuania

## Abstract

Crises in the medicine sector such as the COVID-19 pandemic encourage the search for effective solutions for the provision of health care services, when conventional face-to-face consultations may be difficult to deliver effectively due to contact restrictions. The main objective of this study was to investigate consultation management provided by physicians during the COVID-19 pandemic in Lithuania. The dependence of diagnostic testing and vaccination of patients on the socio-demographic characteristics of physicians was also assessed. An anonymous survey was carried out during the COVID-19 pandemic, between 21 June 2021 and 17 September 2021, involving 191 physicians (9% of the total population) working in family physician teams in Lithuania. Thirty-nine Lithuanian Primary Health Care Institutions (PHCIs) were selected for this study, of which 11 were public and 28 were private. Private and public PHCIs employed 31% and 63% of the respondents, respectively, and 6% of respondents worked at both types of institutions. Concerning telemedicine, the physician-respondents frequently provided consultations over the telephone (79.6%) and in-person (63.9%), but less so via the Internet, with the latter option never being used at all by 57.1% of the respondents. Whilst telephone consultations were frequently provided by Lithuanian physicians, only half of the respondents chose to provide services over the Internet. Private, smaller, and rural-based PHCIs should more actively offer viral diagnostics and vaccination services.

## 1. Introduction

Twenty-first-century challenges such as climate change, the depletion of natural resources, COVID-19 and wars have impacted the economic, medicine and public health, food, science, and innovation sectors, as well as the psychological and mental health of the population [1]. It is known that the COVID-19 pandemic accelerated concerns related to financial issues, fear of death, social isolation, and economic lockdown [1]. In Italy, the results of one study showed that the main fears of the population regarding COVID-19 involved possible vaccine consequences, a state of isolation, and the disease and its consequences [1]. The COVID-19 pandemic dramatically changed the behavior of the population (lifestyle habits, social relationships, mobility, etc.) [1] and raised challenges relating to consultation management in the medicine sector. Both the workload of family physicians and the satisfaction of patients are directly impacted by the growing demand for family physicians’ consultations. Therefore, this gives rise to the search for new means of consulting with patients [2]. Alternative forms of consultation were inevitably sought during the COVID-19 pandemic, with telephone and video consultations being the most common [3]. Physicians at the primary health care level indicated experiencing an increased workload during the pandemic (reporting a NASA-TLX test score of 66.1%, compared to a pre-pandemic level of 48.6%), and the introduction of an e-consultation system provided the means to manage this workload [4]. One study found that introducing video consultations in a family medicine practice made communication between family physicians, medical specialists, and patients easier [5]. Although access to virtual medical consultations improves patient satisfaction, certain challenges in service provision must be addressed. There is, for example, the challenge of patient anonymity and data confidentiality, the assessment and physical examination of a patient’s condition, and the technological skills required of patients to make full use of online tools [6]. Pre-pandemic, teleconsulting was seldom encountered at the primary health care level in any country, but research suggests that the quality of service does not diminish when provided remotely, and patients are satisfied with such methods of communication [7]. Patients exhibiting COVID-19 symptoms can be safely consulted remotely (via telephone or video call) by a general practitioner [8]. The number of consultations provided by family physicians regarding new illnesses decreased during the COVID-19 pandemic, and this impacted the detection rate of new chronic conditions (such as cancer) [5]. One study comparing pre-pandemic and pandemic-era services found that telephone consultations at the primary health care level rose by 122% during the pandemic, whilst video consultations constituted 41.2% of total consultations, compared to a pre-pandemic rate of 19.3% [9]. The same study reported that 40.1% of respondents wished to retain the availability of telephone consultations beyond the pandemic, and 21.9% agreed that they would also like to keep virtual consultations [9]. Another study found that virtual services constituted an average of 66.4% of total services provided by physicians during the COVID-19 pandemic, as opposed to 6.5% pre-pandemic [6]. Family physicians constituted the majority of health care specialists who directly undertook managing patient flows (from diagnostics to referrals) over the course of the pandemic. Based on the above information, the aim of this study was to investigate the purposes and tools of consultation management during the COVID-19 pandemic for physicians working in family physician teams in Lithuania. Specifically, the study addressed physicians’ aims and tools, as well as their approach to diagnostic testing and vaccination. The authors of this study wanted to address the following research question: What were the main reasons for patients seeking care from family physicians, and do they differ depending on physicians’ sociodemographic characteristics? The authors identified this research gap based on insufficient information on the provision of remote services (such as the main goals of consultations and the tools of consultation that were used) in primary health care during the COVID-19 pandemic in Lithuania.

## 2. Theoretical Background

Having first emerged in the 1950s, telemedicine has become a key fixture in health care services in recent years [10]. Advances in communication technologies allow health care providers to engage with patients remotely, and telemedicine is an essential tool in pursuit of this goal [11]. Telemedicine involves health-related communication via the Internet [12], and is defined as the use of information and communication technologies (telephones, computers, etc.) for the purpose of providing health care services [11]. E-consultation is a recent development in the arena of telemedicine services (video calls, telephone consultations, pre-recorded messages, the use of email, etc.) [2]. Telehealth is a broader concept of telemedicine, referring to the use of information technologies to collect patient data [13]. Whilst telemedicine services were once used most frequently by radiologists, cardiologists, and psychiatrists [13], the COVID-19 pandemic saw a surge in demand for remote consultations among family physicians [12]. The adoption of telemedicine improves patient access to services whilst maintaining physical distance to prevent the spread of infection [13]. The most notable challenges of remote service provision include: payment for services rendered; patient safety and privacy; physical examination of the patient; and availability and use of technological resources [13]. Despite these challenges, both patients and physicians acknowledge the advantages of telemedicine, including: improved access to health care services; decreased costs incurred by patients and the health care system; the ability to observe disease progression in a patient; and increased patient satisfaction with services [11]. In this study, the dependence of the aims and tools of consultations on respondents’ sociodemographic characteristics was investigated. The researchers aimed to establish how often telemedicine services were used during the pandemic and understand their dependence on the sociodemographic characteristics of respondents.

## 3. The Health Care System in Lithuania

Specialist health care services were the predominant form in Lithuania prior to health care reform. In 1991, the Lithuanian Supreme Council approved the National Health Concept, seeking to restructure health care services in the direction of primary health care and to establish and develop the institution of family health care [14]. Lithuanian health care reform was undertaken in four stages (the first in 2003–2005, the second in 2006–2008, the third in 2009–2011, and the fourth in 2012–2017) and concentrated on the development of outpatient and care services and the optimization of inpatient services [15]. Health policy and its priorities are developed based on the following determinants of the state of public health: 70% environment- and lifestyle-related; 20% genetics; and 10% as a result of medical service actions [16]. Practical health care prioritizes the development of family medicine institution as a means to remedy 75–80% of health care issues [16]. Family physician standards, adopted in 2005, defined the rights, duties, and competences of a family physician, and the role of family medical practice in primary health care provision within the scope of competences of a qualified family physician [17]. The information system for electronic health services and collaborative infrastructure (the E.health system), launched in 2015, was designed to facilitate patient registration, referrals, vaccination schedules, and prescriptions—for health care providers and patients alike [18]. Although contact services constituted the vast majority of medical services in pre-pandemic Lithuania, the uptake of remote services during lockdown confirmed that physicians are more than capable of consulting with other physicians and their patients using remote communication tools, whether they are audio or audiovisual devices or other electronic communication technologies [19]. The authors of this study investigated the experience of Lithuanian family physicians regarding the goals of consultations and the tools used to achieve them during the pandemic. This focus was chosen due to the fact that remote consultations were first approved during the pandemic. After conducting this research, it is possible to determine how the main goals of consultations and the tools used depend on the sociodemographic characteristics of family physicians in Lithuania. It is also possible to identify opportunities for the development of telemedicine, especially in critical medical situations such as pandemics. The theoretical contribution of this study to consultation management is based on the fact that the obtained results reveal the importance of the management of remote consultations (goals and tools) in the work of family physicians.

## 4. Materials and Methods

### 4.1. Total Population

The sample size of this study was representative of the gender, age, and distribution of physicians across various Lithuanian counties. According to the data provided by the Institute of Hygiene, PHCIs and care homes employed a total of 1,903 family physicians and 238 internal medicine physicians at the end of 2020 (see Table 1).

The majority of family physicians in family physician teams (over 50%) were based in the largest Lithuanian cities, namely Vilnius (32%) and Kaunas (23%). Females dominated both family medicine and the overall health care field in Lithuania, constituting 70% and 84.9% of the total number of physicians, respectively [20]. Based on 2021 data from the State Health Care Accreditation Agency under the Ministry of Health, 85.0% family physicians in Lithuania were female and 15.0% were male. The largest proportion (33%) of family physicians in Lithuania were aged 61–70, a quarter (25%) were aged 51–60, and a further quarter (25%) were aged 40 years or under.

### 4.2. Developing the Questionnaire

The study instrument consisted of 17 questions: 5 open-ended and 12 close-ended. A 5-point Likert-type scale (ranging from *strongly disagree* to *strongly agree*) was used in response to the questions designed to assess the respondents’ opinions on challenges to family medicine during the pandemic (Appendix A). The questionnaire included questions on the respondents’ sociodemographic characteristics—gender, age, years of work experience—and the size, location, and form of ownership of the institution they work at. A 5-point Likert scale was used in the questionnaire, and answers were combined into two groups for analysis:

- In Table 2: “more frequently than before the pandemic”; and “less frequently than before the pandemic or the same amount as during the pandemic.”
- In Table 3: “more frequently than before the pandemic”; and “less frequently than before the pandemic or the same amount as during the pandemic.”
- In Table 4: “never or rarely” and “frequently or always.”
- In Table 5, the answer groups were left the same as in the questionnaire—“yes” and “no.”

Table 6 presents an analysis of the question “Did patients receive a COVID-19 vaccination at your PHCI?” based on sociodemographic characteristics (the ownership and location of the PHCI where respondents work, and the gender of respondents).

**Figure 1:**
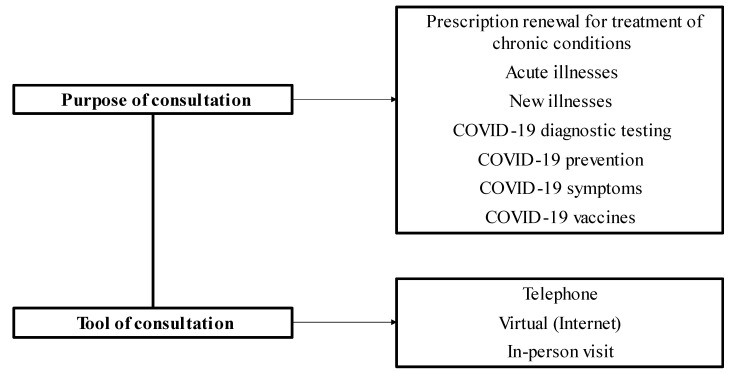
The model for the study of consultations provided by physicians working in teams of family physicians.

In developing the study model, the researchers sought to investigate the main reasons and tools of consultation by family physicians during the COVID-19 pandemic (study model presented in Figure 1).

### 4.3. The Pilot Study

A pilot study took place over 15–30 June 2021, involving 13 physicians from public-owned PHCIs, 9 from private-owned PHCIs, and 1 working at both types of PHCI. The respondents expressed their views on the topic they found of interest, mainly surrounding the workload of physicians and nurses at the primary health care level, the availability of specialist equipment, as well as overtime and salary paid in cases of unlimited work hours.

### 4.4. The Quantitative Study

Before commencing the study, the researchers intended to invite an equal number of physicians from private and public PHCIs (the 50/50 principle), and used selection criteria based on the age and gender of physicians (see Table 2).

However, physicians from public PHCIs participated more actively in the study (see Table 1). Invitations were emailed to the managers and administrators of PHCIs. Participation was voluntary and without remuneration. Upon receipt of an affirmative response, the participants were issued study questionnaires and informed consent forms, both of which were collected 1–8 weeks later by the lead researcher.

### 4.5. Ethics Approval

On 15 June 2021, Kaunas Regional Committee of Biomedical Research Ethics issued permission (No. BE-2-63) to conduct this study.

### 4.6. Statistical Analysis

Data were analyzed using the IBM SPSS Statistics 27 software. The criterion of independence (homogeneity) of c^2^ features and a *z*-test with adjusted *p*-values (Bonferroni method) for pairwise comparisons were used for the analysis of qualitative data. The results were presented as frequency (*n*) and relative frequency (%) of the values of the variables in the groups compared. The quantitative variables did not satisfy the conditions of normal distribution and were, therefore, compared in groups using the Mann–Whitney criterion for non-normally distributed data. The results were presented in medians and quartiles (Q_1_–Q_3_) of the variable. The *z*-test with Bonferroni adjustment was used for chi-square pairwise comparisons. The observed differences were considered statistically significant if the calculated *p*-value was below 0.05.

## 5. Results

### 5.1. Sample Population

A total of 398 questionnaires were distributed for the purposes of this study, of which 191 satisfied the inclusion criteria and 4 were declared invalid, resulting in a 48% response rate (9% of the total population). The characteristics of the study population are presented in Table 3.

### 5.2. Purpose of Consultation

The respondents were asked about the purpose of patient consultations during the COVID-19 pandemic. The responses of family physicians indicated that, compared to before the pandemic, patients contacted them more frequently during the lockdown for the following reasons: COVID-19 testing (*n* = 142; 74.3%); COVID-19 prevention (*n* = 126; 65.97%); and COVID-19 symptoms (*n* = 152; 79.6%). Additionally, nearly all respondents observed that the frequency of patients seeking consultations regarding COVID-19 vaccines increased during lockdown (*n* = 159; 83.2%). Meanwhile, most respondents (*n* = 124; 64.9%) noted no obvious difference in the frequency of consultations for prescription renewals. Concerning the demographic variables of the family physicians who reported observing an increase in consultations for COVID-19 testing and those reporting observing a similar or lower frequency, the χ^2^ analysis revealed statistically significant differences in the duration of employment in their current place of work (*p* = 0.014), number of years of work experience (*p* = 0.012), and age (*p* = 0.02) (see Table 4).

Concerning the demographic variables of the family physicians who reported observing an increase in consultations for COVID-19 testing and those reporting observing a similar or lower frequency, the χ^2^ analysis demonstrated an inconsistent gender-based distribution, with females having a more observable increase than males (see Table 5).

### 5.3. Tools of Consultation

Respondents were asked about the frequency of consultations provided over the telephone, the Internet, or in-person (available choices: *never*, *rarely*, *frequently*, and *always*). Most respondents emphasized frequent telephone (*n* = 152; 79.6%) or in-person (*n* = 122; 63.9%) consultations, whilst a large portion reported never consulting patients over the Internet (*n* = 109; 57.1%). Having observed an irregular distribution of responses in search of links between the sociodemographic characteristics of respondents and tools of consultation, two groups were identified for comparison: those using the indicated tools (1) never or rarely, and (2) frequently or always. Via χ^2^ analysis, statistically significant differences were revealed in the years of work experience (*p* = 0.015) and age (*p* = 0.028) of respondents from the two groups (see Table 6), with younger physicians (in age and years of experience) reporting more *frequently* or *always* responses.

### 5.4. COVID-19 Diagnostic Testing

Responses to the question “*Does your health care institution engage in COVID-19 diagnostic testing?*” were affirmative among the majority of the respondents (*n* = 136; 71.2%). Further analysis revealed a non-homogenous distribution of answers based on the location and form of ownership of the respondents’ place of work (Table 7).

Statistically significantly more urban-based and public-owned PHCI respondents confirmed than denied the statement. Pairwise comparisons of the χ^2^ criterion revealed no statistically significant differences among respondents from large institutions (50–250 employees), but these differences were present among other groups.

### 5.5. COVID-19 Vaccines

Just over half of all physicians (*n* = 110; 57.6%) responded affirmatively to the question “*Does your health care institution administer COVID-19 vaccines?*” The distribution of responses based on the institution’s form of ownership, location, and the respondents’ gender was non-homogenous (see Table 8).

Statistically significantly more female, urban-based, and public-owned PHCI respondents confirmed than denied the statement.

## 6. Discussion

*Purpose of consultation*

Societal behavior is changing in the face of 21st-century crises, and the purposes of personal health care services, whether they concern new or acute illnesses or the treatment of chronic conditions, must be reassessed in view of this [1]. For example, compared to pre-pandemic levels, the number of consultations at the primary care level decreased by 49% over the course of the COVID-19 pandemic [21]. The e-consultation approach to family medicine ensures effective, albeit virtual, physician–patient communication (about vaccines and general safety during a pandemic) when an in-person physical examination of a patient may not be possible [22,23]. This study found the main reasons for requesting a family physician consultation during the pandemic were: consultation regarding the COVID-19 vaccine (83.2%); COVID-19 symptoms (79.6%); testing for COVID-19 (74.3%); COVID-19 prevention (65.97%); and renewing prescriptions for the treatment of chronic conditions (64.9%). Diagnostic testing for COVID-19 was more often performed at public and large PHCIs, and urban-based PHCIs performed COVID-19 tests more often than rural-based PHCIs. The same is true of vaccination rates: they were higher at public-owned and urban-based PHCIs. Female respondents more frequently noted greater COVID-19 vaccination rates and a higher number of consultations concerning COVID-19 prevention among their patients compared to male respondents. Regular requests by patients for COVID-19 testing were noted more frequently among younger, less experienced respondents and those with a shorter length of employment at their current PCHI. Elsewhere, one study observed that consultations for adverse reactions to medication experienced the most significant reduction in number (50.5%), with the highest increase being recorded for issues relating to employment/unemployment during the pandemic (90.2%) [21]. A separate study found that family physicians issued more prescriptions during in-person consultations than via video, and further research is needed to identify the criteria which family physicians apply to prescribing medicine via e-consultations [24].

*Tool of consultation*

Any attempt at improving access to health care services during a major crisis must consider patients’ ability to use technologies for virtual consultations. The majority of older residents and those based in rural areas lack both knowledge of and access to online health care services. Therefore, telephone and in-person consultations must be made available. As evidenced by recent events, the COVID-19 pandemic altered the forms of service provision by family physicians, and remote consulting (via telephone or video conferencing) became the dominant form in many countries. The overall number of consultations by family physicians and specialists for patients with chronic non-infectious conditions decreased during the pandemic, highlighting the importance of remote consulting as a means to ensure continued patient care [25,26]. Family physicians perceive video consultations as superior to telephone consultations, and advocate for retaining them post-pandemic [27]. Patients who made use of remote medical services during the pandemic would prefer to retain such access in the future: 80% desired telephone consultations, and 69% would like to maintain access to video links [28]. The results of this study show that during the pandemic physicians working in family physician teams most frequently consulted patients via telephone (79.6%) or in-person (63.9%), but less so via the Internet, with the latter option never being used at all by 57.1% of respondents. In-person consultations were more frequently provided by younger respondents with fewer years of work experience. However, the tool of remote consultations posed challenges of its own for users on both sides, including: physician–patient communication; technological solutions for service provision; and remote diagnosis of new conditions. Conversely, relevant society-wide education regarding the use of remote services should be introduced as a contingency for unforeseen circumstances (another pandemic, for example). Despite technical malfunctions (patchy Internet service or a lack of relevant technological skills among patients), the introduction of video consultations in family medicine during the COVID-19 pandemic contributed to lower levels of burnout and greater job satisfaction among physicians [29]. Based on previous research, the authors of this study similarly proposed that the broader application of telemedicine services in family medicine could improve emotional wellbeing and job satisfaction among physicians, and lessen professional burnout [30].

*Originality of research*

In Lithuania, family medicine is the main branch of medical practice and science, where the majority of diagnostic and treatment issues are resolved. Whilst the benefits and challenges of remote consultations have been much debated at the practical and theoretical level in recent years, other sectors of society (e.g., education and training, counseling), have embraced remote consultations—no longer as a last resort in difficult times, but as a routine, and, in some cases, preferable, tool of communication. Meanwhile, remote consultations in medicine were legalized in Lithuania only at the onset of the pandemic in 2020, and thus provided researchers with an opportunity to investigate the experience of family physicians in consultation management (goals and tools) during the pandemic. The key findings of this analysis were the relationships between the sociodemographic characteristics of the respondents and the management of the services provided. The key recommendation of this study is that family physicians working in private, smaller, rural PHCIs should be more active in providing diagnostic and vaccination services to their patients.

*Strengths and weaknesses of the study*

This study assessed the self-reported answers of physicians on the tools and purposes of consultations during the COVID-19 pandemic. The key limitation of the study concerns the sample population, i.e., family physicians from a single European country, sharing their opinion about service provision at the primary care level during the COVID-19 pandemic. As such, future studies ought to include secondary- and tertiary-level health care specialists. Furthermore, the opinion of patients is of equal value, and their personal experience with remote consultation could be instrumental in developing training courses aimed at teaching the wider public how to effectively use the new tools of consultation made available since the pandemic.

*Future prospects*

Studies have shown that 21st-century challenges (pandemics, wars, climate change, and the depletion of natural resources) are increasingly the cause of psychological issues among members of society, manifesting in stress, anxiety, depression, and lifestyle changes. The research team behind this article invites others to engage in further studies that consider how changes in public health affect health care service provision in the following fields: the purposes and tools for consultation provision; the introduction of new positions (e.g., crisis management specialists, psychological welfare specialists) in primary health care; and the application of innovations and technological advancements in medicine. Equally, staff must be properly trained in order to fully benefit from innovations (e.g., new forms of service provision, new software) that improve public health and access to health care services in critical times (e.g., wars or pandemics). Future research should reflect the current developments in public health care solutions and introduce practical recommendations to health care policy-makers.

## 7. Conclusions

This study provides an insight into the pandemic-period tools and purposes of physician consultations, and offers a significant contribution towards improving consultation management. The key findings suggest that private, smaller, and rural-based PHCIs should more actively offer viral diagnostics and vaccination services. Similarly, experienced physicians in family physician teams should offer viral testing on a regular basis. Meanwhile, telemedicine services were commonly used in other countries during the pandemic and contributed to lower exhaustion rates and increased job satisfaction. The core message for policy makers is that the majority of physicians and patients would prefer to retain the use of remote services post-pandemic; therefore, health care systems should embrace remote service provision and secure sufficient funding for its development in the future.

## Figures and Tables

**Table 1:** The distribution of family physicians in Lithuanian counties.

County	Number of Family Physicians (*N*), Total	Number of Internal Medicine Physicians (*N*), Total	Number of Family and Internal Medicine Physicians in the County (*N*), Total
Vilnius	596	87	683
Kaunas	452	40	492
Klaipėda	203	14	217
Šiauliai	167	14	181
Panevėžys	118	23	141
Utena	79	13	92
Marijampolė	84	14	98
Tauragė	48	7	55
Telšiai	80	9	89
Alytus	76	17	93
**Total**	**1903**	**238**	**2141**

Note: The Institute of Hygiene, end of 2020 data.

**Table 2:** The distribution of family physicians in the study population based on age and gender.

Distribution Based on Age and Gender		Total Number of Family Physicians (*N*), Total	Relative Distribution Based on Age and Gender, % of Total Study Population (*N)*	Percentage of Age Group, %	Estimated Number of Physicians to be inCluded in the Survey (*N* = 398)
Under 40	Male	119	13	25	13
	Female	808	87		87
**Total**		**927**	**100**		**100**
41–50	Male	69	17	10	7
	Female	336	83		32
**Total**		**405**	**100**		**39**
51–60	Male	147	16	25	16
	Female	792	84		84
**Total**		**939**	**100**		**100**
61–70	Male	181	15	33	20
	Female	1054	85		111
**Total**		**1235**	**100**		**131**
71+	Male	52	18	7	5
	Female	231	82		23
**Total**		**283**	**100**	**100**	**28**

Note: State Health Care Accreditation Agency under the Ministry of Health, 2021 data.

**Table 3:** The characteristics of the study population.

Variable		Frequency	
		Frequency (*n*)	Relative Frequency (%)
Location	Urban area	169	88.5
	Rural area	22	11.5
Gender	Female	161	84.3
	Male	30	15.7
Size of institution	Very small and small (<=50)	70	36.6
	Medium (50–250)	53	27.7
	Large (>250)	68	35.6
Form of ownership of institution	Private	59	30.9
	Public	120	62.8

**Table 4:** The relationship between the sociodemographic characteristics of physicians and purpose of consultation being COVID-19 diagnostics.

Characteristic	Purpose of Consultation: COVID-19 Diagnostic Testing during the Pandemic		*p*
	More Frequently than Pre-Lockdown (*n* = 142)	Similarly or Less Frequently than Pre-Lockdown (*n* = 49)	
Duration of employment (years)	11.5 (3–30)	19 (8–40)	0.014
Years of work experience	25 (9.75–38)	36 (15–43.5)	0.012
Age	53.5 (37–63)	61 (38.5–69.5)	0.02

Note. Data presented as a median (Q_1_–Q_3_).

**Table 5:** The gender-based distribution of respondents indicating that the purpose of consultation was COVID-19 prevention.

Characteristic	Purpose of Consultation: COVID-19 Prevention		*p*
	More Frequently than Pre-Lockdown, *n* (%)	Similarly or Less Frequently than Pre-Lockdown, *n* (%)	
Female	111 (88.1)	50 (76.9)	0.044
Male	15 (11.9)	15 (23.1)	

**Table 6:** Relationships between the sociodemographic characteristics of the respondents and the in-person toolof consultation.

Characteristic	In-Person Consultation with a Patient		*p*
	Never or Rarely (*n* = 57)	Frequently or Always (*n* = 134)	
Years of work experience	35 (18–40.50)	22.50 (8–38.25)	0.015
Age (years)	60 (48.5–68)	52.5 (37–63.25)	0.028

Note. Data presented as a median (Q_1_–Q_3_).

**Table 7:** Relationships between the sociodemographic characteristics of respondents and diagnostic testing for COVID-19.

Characteristic		Does Your Health Care Institution Engage in COVID-19 Diagnostic Testing?		*p*
		Yes *n* (%)	No *n* (%)	
Institution’s form of ownership	Private	33 (26.2)	26 (49.1)	0.003
	Public	93 (73.8)	27 (50.9)	
Size	Up to 50 employees	37 (27.2) ^a^	33 (60.0) ^a^	<0.001
	50–250 employees	43 (31.6)	10 (18.2)	
	Over 250 employees	56 (41.2) ^b^	12 (21.8) ^b^	
Location	Urban	126 (92.6)	43 (78.2)	0.005
	Rural	10 (7.4)	12 (21.8)	

Note. Pairwise comparisons a-a and b-b of χ^2^ criterion (*z*-test); *p* < 0.05.

**Table 8:** Relationships between the sociodemographic characteristics of respondents and COVID-19 vaccine administration.

Characteristic		Does Your Health Care Institution Administer COVID-19 Vaccines?		*p*
		Yes *n* (%)	No *n* (%)	
Institution’s form of ownership	Private	22 (21.6)	37 (48.1)	<0.001
	Public	80 (78.4)	40 (51.9)	
Gender	Female	98 (89.1)	63 (77.8)	0.034
	Male	12 (10.9)	18 (22.2)	
Location	Urban	93 (84.5)	76 (93.8)	0.047
	Rural	17 (15.5)	5 (6.2)	

## Data Availability

The datasets used and analyzed during this study are available from the corresponding author on reasonable request.

## Appendix A

**Questionnaire for the “Challenges in Family Medicine Posed by the COVID-19 Pandemic” study**

1. Where is your family medical institution located?
  (a) In an urban area
  (b) In a rural area
2. Which type of primary health care institution are you employed in?
  (a) Private
  (b) Public
  (c) Both private and public
3. Select the box that best represents your opinion of the corresponding statement.

Mark your answers with a ˅.

**Statement**	**Strongly Disagree**	**Disagree**	**Neither Agree Nor Disagree**	**Agree**	**Strongly Agree**
The COVID-19 pandemic concerns me					
The COVID-19 pandemic negatively affected my quality of life					
I feel burned out during the COVID-19 pandemic					
I am satisfied with the management of the COVID-19 pandemic in the country					

4. How frequently do you encounter the following purposes of consultation compared to before and during the COVID-19 quarantine?

Mark your answers with a ˅.

**Purpose of consultation**	**More Frequently Than Before the Pandemic**	**Less Frequently Than Before the Pandemic**	**The Same Amount as during the Pandemic**
Consultation regarding the renewal of prescriptions to treat chronic diseases			
Consultation regarding COVID-19 prevention			
Consultation regarding the symptoms caused by the COVID-19 virus			
Consultation regarding the diagnostics of the COVID-19 virus			
Consultation regarding the COVID-19 vaccine			
Consultation regarding an acute illness			
Consultation regarding a new illness			

5. How often do you use the following tools to consult with your patients during the COVID-19 pandemic?

Mark your answers with a ˅.

**Tool of Consultation**	**Never**	**Rarely**	**Often**	**Always**
Telephone				
Online				
In-person				

6. Select the box that best represents your opinion of the corresponding statement.

Mark your answers with a ˅.

**Statement**	**Strongly Disagree**	**Disagree**	**Neither Agree Nor Disagree**	**Agree**	**Strongly Agree**
My patients are concerned by the COVID-19 pandemic					
The COVID-19 pandemic is negatively affecting the quality of life of my patients					
My patients feel burned out during the COVID-19 pandemic					
My patients are satisfied with the management of the COVID-19 pandemic in this country					

7. Select the box that best represents your opinion of the corresponding statement.

Mark your answers with a ˅.

**Statement**	**Strongly Disagree**	**Disagree**	**Neither Agree Nor Disagree**	**Agree**	**Strongly Agree**
My work satisfaction has decreased during the COVID-19 pandemic					
My patients experienced reduced access to family medicine services during the COVID-19 pandemic					
The COVID-19 pandemic is a threat to my own life and safety, as well as that of my colleagues					
The satisfaction of my patients with family medicine services has decreased during the COVID-19 pandemic					
New tools are required to provide ordinary consultations during the COVID-19 pandemic					

8. Does the institution you work for offer COVID-19 vaccines?
  (a) Yes
  (b) No
9. Does the institution you work for perform diagnostic laboratory tests for COVID-19?
  (a) Yes
  (b) No
10. How far do you agree that the following aspects of the COVID-19 pandemic were exhausting?

Mark your answers with a ˅.

**Statement**	**Strongly Disagree**	**Disagree**	**Neither Agree Nor Disagree**	**Agree**	**Strongly Agree**
Constantly changing laws					
Incomprehensive nature of remote consultations					
Chaotic vaccination priorities					
Patients’ fear of attending consultations					
Large workload					
Unsatisfied patients					
Malfunction of online systems					
Other reasons for exhaustion (please specify)					

11. How many employees work in your institution?
  (a) 0–10
  (b) 11–50
  (c) 51–250
  (d) 251+
12. How long have you been working for your current employer? (Please specify a number) ___________________
13. How many years of work experience do you have? (Please specify a number) ______________________
14. What is your gender?
  (a) Female
  (b) Male
15. How old are you? (Please specify a number) _______________________________________
16. To which Lithuanian municipality does the institution where you work belong? (Write your answer)_________________________________________________________________________
17. Please provide recommendations on how to more effectively manage the services provided by family medical institutions during the COVID-19 pandemic. ________________________________________________________________________________________________________________________________________________________________________________________________________________________________________________________________________________________________________________________________________________________________________________________________________________________________________________________________________________________________________________________________________________________________________________________________________________________________________________________________________________________________________________________________

Thank you for taking the time to complete this questionnaire.
